# Biosynthesis, Characterization, and Augmented Anticancer Activity of ZrO_2_ Doped ZnO/rGO Nanocomposite

**DOI:** 10.3390/jfb14010038

**Published:** 2023-01-09

**Authors:** Maqusood Ahamed, Rashid Lateef, M. A. Majeed Khan, Pavan Rajanahalli, Mohd Javed Akhtar

**Affiliations:** 1 Department of Physics and Astronomy, College of Science, King Saud University, Riyadh 11451, Saudi Arabia; 2 Department of Biochemistry, Faculty of Science, Veer Bahadur Singh Purvanchal University, Jaunpur 222003, Uttar Pradesh, India; 3 Department of Biology, University of Tampa, Tampa, FL 33569, USA

**Keywords:** green synthesis, ginger extract, ZnO/ZrO2/rGO, cancer therapy, biocompatibility

## Abstract

Fabrication of ZnO nanoparticles (NPs) via green process has received enormous attention for its application in biomedicine. Here, a simple and cost-effective green route is reported for the synthesis of ZrO_2_-doped ZnO/reduced graphene oxide nanocomposites (ZnO/ZrO_2_/rGO NCs) exploiting ginger rhizome extract. Our aim was to improve the anticancer performance of ZnO/ZrO_2_/rGO NCs without toxicity to normal cells. The preparation of pure ZnO NPs, ZnO/ZrO_2_ NCs, and ZnO/ZrO_2_/rGO NCs was confirmed by transmission electron microscopy (TEM), scanning electron microscopy (SEM), energy dispersive X-ray spectroscopy (EDS), X-ray diffraction (XRD), photoluminescence (PL), and dynamic light scattering (DLS). XRD spectra of ZnO/ZrO_2_/rGO NCs exhibited two distinct sets of diffraction peaks, ZnO wurtzite structure, and ZrO_2_ phases (monoclinic + tetragonal). The SEM and TEM data show that ZrO_2_-doped ZnO particles were uniformly distributed on rGO sheets with the excellent quality of lattice fringes without alterations. PL spectra intensity and particle size of ZnO decreased after ZrO_2_-doping and rGO addition. DLS data demonstrated that green prepared samples show excellent colloidal stability in aqueous suspension. Biological results showed that ZnO/ZrO_2_/rGO NCs display around 3.5-fold higher anticancer efficacy in human lung cancer (A549) and breast cancer (MCF7) cells than ZnO NPs. A mechanistic approach suggested that the anticancer response of ZnO/ZrO_2_/rGO NCs was mediated via oxidative stress evident by the induction of the intracellular reactive oxygen species level and the reduction of the glutathione level. Moreover, green prepared nanostructures display good cytocompatibility in normal cell lines; human lung fibroblasts (IMR90) and breast epithelial (MCF10A) cells. However, the cytocompatibility of ZnO/ZrO_2_/rGO NCs in normal cells was better than those of pure ZnO NPs and ZnO/ZrO_2_ NCs. Augmented anticancer potential and improved cytocompatibility of ZnO/ZrO_2_/rGO NCs was due to ginger extract mediated beneficial synergism between ZnO, ZrO_2_, and rGO. This novel investigation emphasizes the significance of medicinal herb mediated ZnO-based NCs synthesis for biomedical research.

## 1. Introduction

Cancer is one of the primary causes of death globally, with an anticipated 19 million (M) new cases and about 10 million mortalities due to cancer in the year 2020. The most common types of cancer are breast (2.26 M cases), lung (2.21 M cases), colon and rectum (1.93 M cases), prostate (1.41 M cases), and skin (120 M cases) [1]. Current chemotherapies are insufficient due to the development of drug resistance, non-selectivity for neoplastic tissue, and the requirement of high dosages [2]. Recent developments in nanomedicine are anticipated to improve cancer treatment by increasing the drug efficacy and reducing the toxicity to non-cancerous cells [3]. The nanoscale form of ZnO has grabbed worldwide attention for cancer treatment because of its tunable physicochemical properties. Nanoscale ZnO is an important semiconductor with unique characteristics, e.g., high piezoelectricity, wide band gap, and large excitation binding energy. The bulk form of ZnO is also listed as “Generally Recognized as Safe” (GRAS) by the US food and drug authority.

Several investigations observed that ZnO nanoparticles (NPs) exert selective toxicity toward tumor cells, causing fewer effects on normal cells. For example, earlier we observed that ZnO NPs induce cytotoxicity in human alveolar adenocarcinoma (A549) and hepatocellular carcinoma (HepG2) cells, and fewer effects on primary rat hepatocytes [4]. Another study found that ZnO NPs exhibited selective cytotoxicity in a number of human glioma cells, with minimal effects on normal astrocytes [5]. A current work found that ZnO NPs display antitumor activity in human small cell lung cancer (SCLC) in orthotopic mice [6]. Conversely, some studies demonstrate the toxicity of ZnO NPs in different organisms including marine organisms, zebrafish, and mammalian models [7,8,9]. This shortcoming can restrict the application of ZnO NPs in cancer therapy. Hence, there is an urgent need for a novel method to enhance the anticancer efficacy of ZnO NPs without many side effects.

The preparation of ZnO-based nanocomposites (NCs) is now providing new tools for cancer research [10,11,12]. Graphene derivatives such as reduced graphene oxide (rGO) are considered wonder nanostructures because of their exceptional characteristics, e.g., surface defects, high surface area, and presence of several surface oxygen functional groups [13]. Ziconium oxide (ZrO_2_) possesses plenty of oxygen vacancies on its surface, with redox activity that makes it a suitable candidate for biological activities [14]. These properties of ZrO_2_ and graphene derivatives provide favorable conditions for the preparation of ZnO and graphene derivatives-based NCs that can be utilized in biomedicine [15]. Recently, metal oxide–graphene derivatives-based NCs are being synthesized because of their superior characteristics that cannot be attained by individual material [16]. Several physical methods are being used for the synthesis of ZnO-based NCs [17,18]. However, physical routes are costly and requires high energy and high temperature, whereas chemicals methods release hazardous chemicals into the environment. Moreover, nanostructures prepared by chemical and physical routes are not suitable for biomedical research [19]. Recently, researchers have been utilizing cost-effective and environmentally friendly methods for the preparation of ZnO-based NCs than can be applied in cancer therapy [20,21].

In this investigation, ZnO NPs, ZnO/ZrO_2_ NCs, and ZnO/ZrO_2_/rGO NCs were synthesized by a simple, cost-effective, and eco-friendly method using ginger (*Zingiber officinale*) rhizome extract. The intention was to assimilate the valuable characteristics of ZnO, ZrO_2_, and rGO into a nanocomposite with augmented anticancer efficacy and without side effects. Ginger has been generally used as a household spice and herbal medicine for a long time [22]. Ginger extract has a wide range of bioactive phytochemicals, such as gingerol, shogaol, paradols, alkaloids, and flavonoids [23]. Due to the presence of these phytochemicals, ginger has shown a number of activities such as antimicrobial, anticancer, and anti-inflammatory activities [22,24]. Bioactive phytochemicals of ginger act as reducing and stabilizing components for the preparation of ZnO/ZrO_2_/rGO NCs. Synthesized samples (ZnO NPs, ZnO/ZrO_2_ NCs, and ZnO/ZrO_2_/rGO NCs) were characterized by photoluminescence (PL), field emission scanning electron microscopy (FESEM), X-ray diffraction (XRD), field emission transmission electron microscopy (FETEM), and energy dispersive spectroscopy (EDS) techniques. Anticancer efficacy of synthesized samples was explored in human breast (MCF7) and lung (A549) cancer cells. The potential mechanism of anticancer response of these nanostructures was studied through oxidative stress. The cytocompatibility of green synthesized nanostructures was examined in normal breast epithelial (MCF10A) and lung fibroblast (IMR90) cell lines. Human lung and breast cancer cells were chosen in this investigation, as these cancers are currently found most commonly worldwide [1].

## 2. Materials and Methods

### 2.1. Preparation Ginger Rhizome Extract

Fresh ginger rhizome was bought locally. Ginger rhizome was thoroughly rinsed with double distilled water (DDW) to eliminate dust and debris. Washed ginger rhizome was dried (in a food drier) and ground into fine powder. Further, 10 g of ginger powder was soaked in 500 mL of DDW and stirred on a hot plate at 100 °C for 1 h. Then, the mixture was cooled to room temperature. At last, the mixture was filtered to produce a light-yellow clear solution (ginger extract) that was stored at 4 °C for further study.

### 2.2. Preparation of ZnO NPs, ZnO/ZrO_2_ NCs, and ZnO/ZrO_2_/rGO NCs

**Figure 1:**
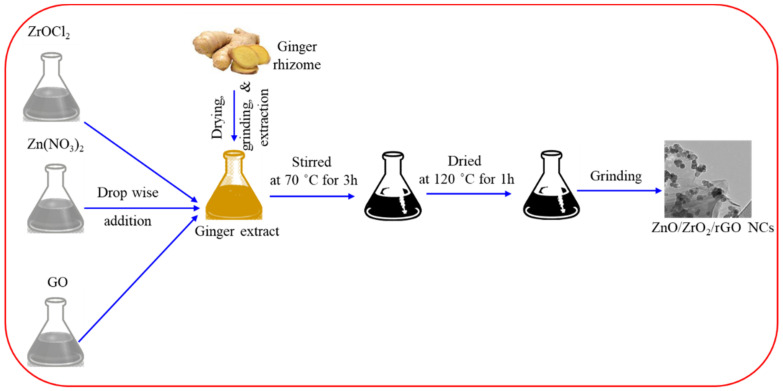
Green preparation of ZnO/ZrO_2_/rGO NCs using ginger rhizome extract.

Zinc nitrate (Zn(NO_3_)_2_.6H_2_O) (CAS No. 10196-18-6, Millipore-Sigma, St Louis, MO, USA), zirconyl chloride (ZrOCl_2_.8H_2_O) (CAS No. 13520-92-8, Millipore-Sigma), and graphene oxide (GO) (Millipore-Sigma) were used as precursors. The 2 mM of zinc nitrate (100 mL) and 0.1 mM of zirconyl chloride (100 mL) were prepared in deionized water in separate beakers. The 0.1 g of GO was also suspended in 100 mL of deionized water with continuous stirring to produce a homogeneous suspension. Then, 50 mL of each solution/suspension (zinc acetate, zirconyl chloride, and GO) were simultaneously added dropwise into 150 mL of ginger extract. The mixture was stirred on a hot plate at 70 °C for 3 h. The resultant mixture was centrifuged, washed with deionized water and desiccated in an oven at 120 °C for 1 h and ground into a fine powder of ZnO/ZrO_2_/rGO NCs. The ZnO/ZrO_2_ NCs were prepared by a similar route, without GO suspension. Moreover, pure ZnO NPs were synthesized by the same method without mixing of zirconyl chloride and GO. Figure 1 represented the schematic of the green preparation of ZnO/ZrO_2_/rGO NCs.

Endotoxin assay is an essential parameter analysis to assess the possible contamination of nanostructures, especially for bio-interaction studies [25]. The chromogenic limulus amebocyte (LAL) assay was applied to examine possible contamination present in prepared nanostructures. Results of this assay showed that green synthesized ZnO NPs, ZnO/ZrO_2_ NCs, and ZnO/ZrO_2_/rGO NCs were devoid of contaminations from endotoxin (Figure S1, Supplementary Information).

### 2.3. Characterization

Structural characterization of prepared samples (ZnO NPs, ZnO/ZrO_2_ NCs, and ZnO/ZrO_2_/rGO NCs) was carried out by X-ray diffraction (XRD) (PANalytical, X’Pert Pro, Malvern Instruments, UK), transmission electron microscopy (TEM) (JEM-2100, JEOL, Inc., Tokyo, Japan), and scanning electron microscopy (SEM) (JSM-7600F, JEOL, Inc.). Elemental composition and elemental mapping were carried out by energy dispersive X-ray spectroscopy (EDS). Photoluminescence (PL) analysis was carried out using a fluorescence spectrophotometer (Hitachi F-4600, Tokyo, Japan). Characterization of the aqueous suspension of green prepared samples was carried out at dynamic light scattering (DLS, ZetaSizer, Nano-HT, Malvern Instruments).

### 2.4. Cell Culture

Human lung cancer cell line (A549, CCL-185), human breast cancer cell line (MCF7, HTB-22), human normal lung fibroblast (IMR90, CCL-186), and human normal breast epithelial cell line (MCF10A, CRL-10317) were obtained from American Type Culture Collection (ATCC, VA, USA). Cells were grown in DMEM (Dulbecco’s Modified Eagle’s Medium) with 10% FBS (fetal bovine serum), 100 µg/mL streptomycin, and 100 U/mL penicillin. Cells were maintained at 37 °C with 5% CO_2_.

### 2.5. Exposure of Cells

A total of 1 mg/mL of stock suspension of each synthesized nanostructure was concocted using de-ionized water. After 30 min of sonication, stock suspension was diluted into appropriate concentrations (1–200 µg/mL) with culture media for exposure to cells. Cells without NP/NCs exposure were selected as controls.

### 2.6. Bioactivity Study

The anticancer performance of synthesized nanostructures was assessed using MTT cell viability assay [26] with some modifications [27]. In this assay, live cells reduce the yellow colored MTT into purple colored fomazan crystals. These crystals were dissolved in isopropanol to produce a clear solution. Absorbance of this solution was recorded at 570 nm utilizing a microplate reader (Synergy-HT BioTek, Winooski, VT, USA). Intracellular level of reactive oxygen species (ROS) generation was estimated with a probe 2′-7′-dichlorodihydrofluorescein diacetate (H_2_DCFDA, Millipore-Sigma) according to our earlier protocol [28]. The non-fluorescent H_2_DCFDA probe changed to highly fluorescent 2′-7′-dicholorofluorescein (DCF) after reaction with ROS. The intracellular fluorescence level of DCF was recorded at 485/520 nm of excitation/emission wavelength with a microplate reader (Synergy-HT, BioTek). Elman’s reagent was used to assess the antioxidant glutathione (GSH) level [29]. Protein content was assayed using Bradford’s protocol [30]. 

### 2.7. Statistical Analysis

Bioactivity results were analyzed by one-way analysis of variance (ANOVA) followed by Dennett’s tests. The *p* < 0.05 was attributed as statistically significant. All bioactivity data were presented as the mean ± SD of three independent tests (*n* = 3).

## 3. Results and Discussion

### 3.1. XRD Analysis

**Figure 2:**
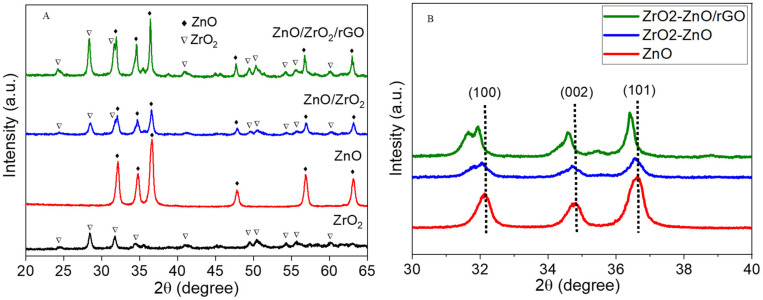
(**A**) XRD spectra of prepared nanostructures. (**B**) Peaks shift.

Figure 2A represented the XRD spectra of synthesized nanostructures. Pure ZnO NPs exhibited sharp and strong peaks at (2θ) 32.12°, 34.82°, 36.64°, 47.79°, 56.87°, and 63.15°, corresponding to (100), (002), (101), (102), (110), and (103) reflections, respectively, and can be indexed with the standard peaks of hexagonal wurtzite crystalline structure of ZnO (JCPDS No. 36-1451) [31]. XRD spectra of pure ZrO_2_ NPs showed a mixture of monoclinic and tetragonal crystalline phases. The peaks at (2θ) 24.34°, 28.45°, 40.95°, 49.45°, 54.28°, and 55.68° corresponding to (110), (111), (120), (220), (003), and (310), respectively, are characteristics of monoclinic crystalline phases (JCPDS No. 37-1484). Moreover, the peaks at (2θ) 31.76°, 34.46°, 50.43°, and 60.09°, corresponding to (011), (110), (112), and (211), respectively, are characteristics of tetragonal crystalline phases (JCPDS No. 79-1771). Both monoclinic and tetragonal crystalline phases of ZrO_2_ NPs were also observed by other investigators [32].

XRD spectra of ZnO/ZrO_2_ NCs and ZnO/ZrO_2_/rGO NCs evidently exhibit two different sets of diffraction peaks: ZnO wurtzite structure, and ZrO_2_ (monoclinic + tetragonal) structure. These spectra suggested the formation of ZnO/ZrO_2_ NCs. The rGO peaks did not appear in ZnO/ZrO_2_/rGO NCs, which suggests the ZnO/ZrO_2_ NCs homogeneously anchored on rGO sheets and prohibited the restacking of sheets. The rGO integration also did not change the crystalline structure of ZnO/ZrO_2_ NCs. Moreover, an obvious shift of the three main peaks of ZnO (100, 002, and 101) to lower 2θ angle was noticed following the integration of ZrO_2_ and rGO, which suggests the successful synthesis ZnO/ZrO_2_/rGO NCs (Figure 2B). These shifts can also be ascribed to the replacement of Zn^2+^ (0.075 nm) by Zr^4+^ (0.072 nm) of lower ionic radii [33]. The particle size of prepared nanostructures was calculated corresponding to the prominent peak (101) using the Debye–Scherrer formula [34]. Results showed that crystallite sizes of ZnO NPs, ZnO/ZrO_2_ NCs, and ZnO/ZrO_2_/rGO NCs were 25, 20, and 13 nm, respectively (Table 1).

### 3.2. TEM Analysis

**Figure 3:**
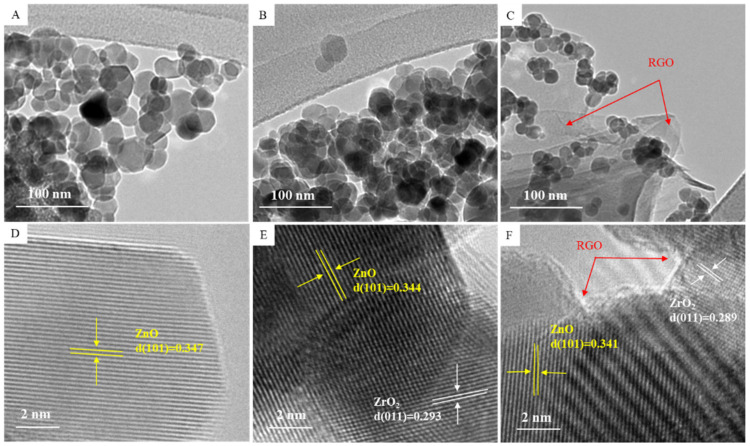
Low-resolution (**A**–**C**) and high resolution (**D**–**F**) TEM micrographs of prepared nanostructures.

**Figure 4:**
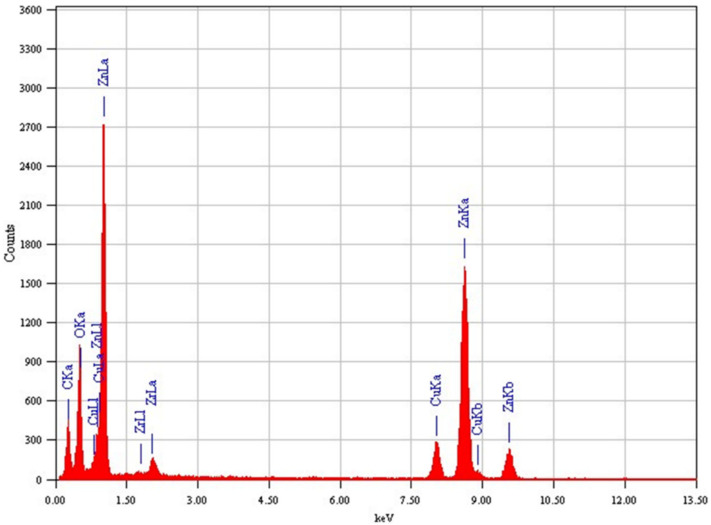
TEM-EDS showed the elemental presence in the ZnO/ZrO_2_/rGO NCs.

The structural characterization of green synthesized samples was further carried out by FETEM. Figure 3A–C showed the low-resolution micrographs of ZnO NPs, ZnO/ZrO_2_ NCs, and ZnO/ZrO_2_/rGO NCs. The morphology of pure ZnO NPs was almost sphere-shaped, with an average size of 23 nm. We noticed that the morphology of the ZnO NPs remained similar, although the size of particles decreases after ZrO_2_-doping (18 nm) and rGO addition (12 nm) (Table 1). Moreover, ZrO_2_-doped ZnO particles were firmly attached on rGO nanosheets and acted as spacers to prevent the restacking of nanosheets that enhance the overall surface area of NCs. The reduction of NPs size following metal oxide doping and rGO incorporation was also observed in earlier studies [35,36]. Smaller size and greater surface area of nanostructures is related to an improved therapeutic efficacy [37,38]. HRTEM micrographs of ZnO NPs, ZnO/ZrO_2_ NCs, and ZnO/ZrO_2_/rGO NCs are presented in Figure 3D–F. These HRTEM micrographs demonstrated that NPs were highly crystalline and there were good synergisms of ZnO, ZrO_2_, and rGO with excellent quality of lattice fringes deprived of distortion. The estimated interplanar spacing of adjacent lattice fringes of ZnO, ZrO_2_-doped ZnO, and ZnO/ZrO_2_/rGO were 0.347, 0.344, and 0.341, respectively, corresponding to the (101) plan of hexagonal wurtzite crystal of ZnO. These results were in agreement with XRD spectra. TEM-EDS analysis of ZnO/ZrO_2_/rGO NCs showed the existence of Zn, Zr, O, and C elements without other elemental contaminations (Figure 4). The presence of the Cu peak was due to the use of the copper grid in TEM measurement. 

### 3.3. SEM Analysis

**Figure 5:**
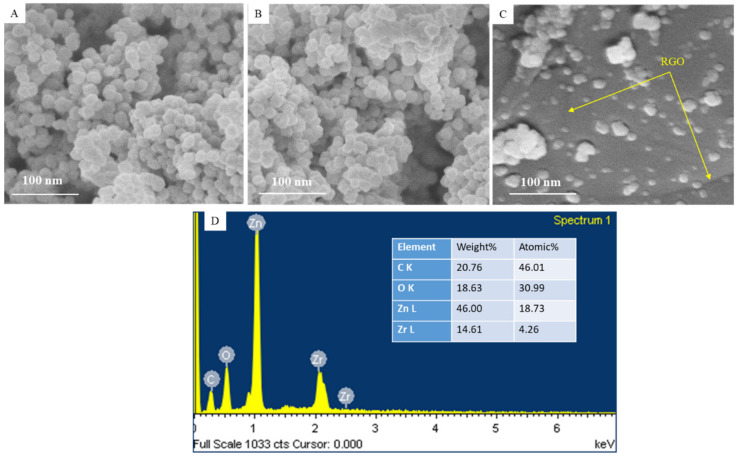
(**A**–**C**) SEM micrographs of prepared nanostructures. (**D**) SEM-EDS showed elemental percentage in the ZnO/ZrO_2_/rGO NCs.

**Figure 6:**
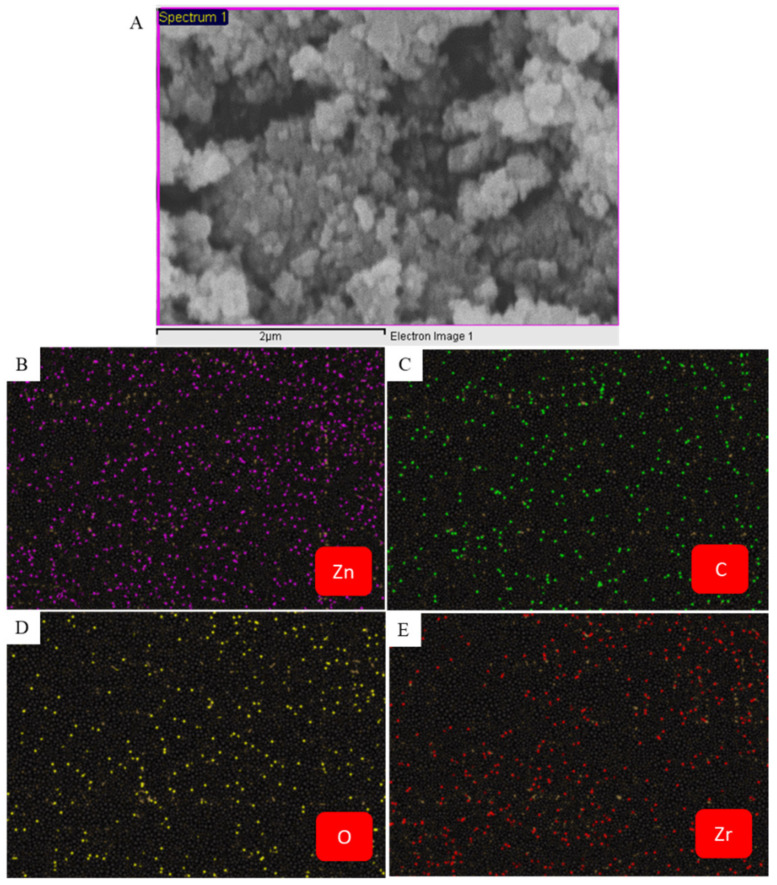
(**A**) Electron micrograph of ZnO/ZrO_2_/rGO NCs. (**B**–**E**) Zn, C, O, and Zr elemental mapping.

Figure 5A–C show the FESEM micrographs of prepared nanostructures. These micrographs indicated the uniform surface morphology of ZnO NPs, and ZrO_2_-doping and rGO assimilation did not alter the particles surface smoothness. Moreover, the FESEM micrograph of ZnO/ZrO_2_/rGO NCs showed that particles were homogenously anchored on rGO nanosheets (Figure 5C). SEM-EDS elemental analysis further confirmed the existence of Zn, Zr, O, and C in ZnO/ZrO_2_/rGO NCs. The presence of Zn, Zr, O, and C in ZnO/ZrO_2_/rGO NCs was further confirmed by elemental mapping (Figure 6).

### 3.4. Photoluminescence Study

**Figure 7:**
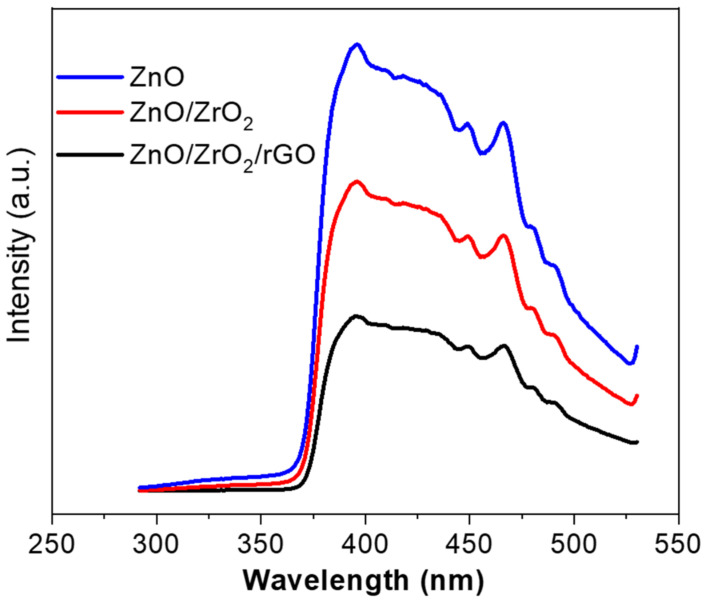
Photoluminescence spectra of green synthesized nanostructures.

At an excitation wavelength of 290 nm, the PL spectra of prepared nanostructures were recorded and shown in Figure 7. These NPs/NCs display photoemission peaks in both the UV and visible regions. The peak at 395 nm represents near band edge emission and the peaks at 448, 466, 480, and 491 nm correspond to blue emission. The visible emission is related to the zinc interstitials, oxygen vacancies, oxygen interstitials, and surface defects of the synthesized samples [39]. The UV emission peak (395 nm) is generated due to the recombination of free exciton between the valence band and the conduction band of ZnO [18]. Peak intensity of ZnO NPs decreased after the addition of ZrO_2_ and rGO, which is credited to the reduced electron-hole recombination. This phenomenon is beneficial in improving the anticancer efficacy of semiconductor nanostructures [40]. The reduction of peak intensities further confirms the synergistic interaction between ZnO, ZrO_2_, and rGO.

### 3.5. DLS Analysis

Hydrodynamic size and zeta potential in culture media were recorded at DLS (Figure S2, Supplementary Materials). Table 1 showed that hydrodynamic size of green prepared ZnO NPs, ZnO/ZrO_2_ NCs, and ZnO/ZrO_2_/rGO NCs was several times higher than size calculated from XRD and TEM. The agglomeration of particles in aqueous medium could be the possible reason for higher hydrodynamic size [38]. Zeta potential data suggest that prepared samples were fairly stable in an aqueous suspension. Moreover, the colloidal stability of ZnO/ZrO_2_/rGO NCs (−29 mV) was better than pure ZnO NPs (−17). Higher colloidal stability of colloidal suspensions is associated with improved biological interaction of NPs/NCs [16,21].

### 3.6. Anticancer Activity

**Figure 8:**
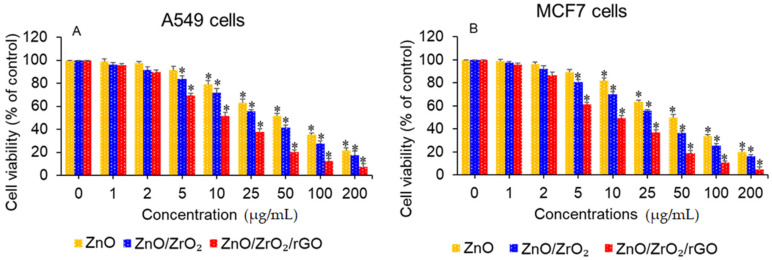
Anticancer activity of green synthesized nanostructures. (**A**) Human lung cancer A549 cells. (**B**) Human breast cancer MCF7 cells. * *p* < 0.05 vs. control.

Recent studies highlight the importance of environmentally friendly fabrications of metal oxide nanostructures for biomedical applications [41]. ZnO NPs possess the intrinsic characteristic of selectively killing cancer cells with less toxicity to normal cells, which can be further enhanced by tailoring its physicochemical characteristics [42]. In this work, anticancer efficacy of green produced ZnO NPs, ZnO/ZrO_2_ NCs, and ZnO/ZrO_2_/rGO NCs was assessed in human cancer cells (A549 and MCF7). Both kinds of cancer cells were treated with different concentration (1–200 µg/mL) of ZnO NPs, ZnO/ZrO_2_ NCs, and ZnO/ZrO_2_/rGO NCs for 24 h, and anticancer potential was examined. Results demonstrated that all three nanostructures induce concentration-dependent cytotoxicity in both A549 and MCF7 cancer cells (Figure 8A,B). The IC_50_ values of ZnO NPs, ZnO/ZrO_2_ NCs, and ZnO/ZrO_2_/rGO NCs are present in Table 2. Anticancer efficacy of ZnO/ZrO_2_/rGO NCs against both A549 and MCF7 cancer cells was almost 3.5 times higher than ZnO NPs. The high anticancer activity of ZnO/ZrO_2_/rGO NCs might be because of the excellent synergism between ZnO, ZrO_2_, and rGO mediated by ginger phytochemicals. There are a number of studies demonstrating the antitumor activity of ginger extracts [22,23]. Tsai et al. observed that 6-Gingerol (a natural phenol present in ginger extract) blocks the proliferation of human lung cancer A549 cells [24]. Another study reported that MCF7 and MDA-MB-231 cells following 6-shogaol (ginger derived bioactive compound) exposure underwent cell death through cell cycle arrest [43]. Therefore, ginger extract mediated green prepared ZnO/ZrO_2_/rGO NCs raise a hope for their therapeutic potential in cancer treatment. Results of this work agreed with earlier reports that observed the improved anticancer efficacy of ZnO-based nanocomposites prepared through a green route [36,44].

### 3.7. Cytocompatibility

**Figure 9:**
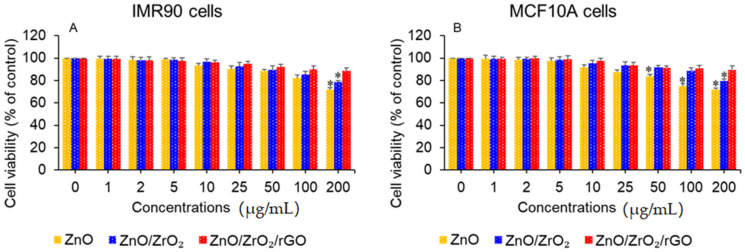
Cytocompatibility of green synthesized nanostructures. (**A**) Human normal lung fibroblasts IMR90. (**B**) Human normal breast epithelial MCF10A. * *p* < 0.05 vs. control.

Selectivity is an important issue for anticancer drugs [45]. Therefore, biocompatibility with normal tissues/cells is an essential feature for newly proposed anticancer agents. In this work, cytotoxicity of green produced nanostructures was also estimated in non-cancerous normal cell lines: human normal lung fibroblasts (IMR90) and human normal breast cancer cells (MCF10A). Both types of human normal cells were treated to different concentrations (1–200 µg/mL) of ZnO NPs, ZnO/ZrO_2_ NCs, and ZnO/ZrO_2_/rGO NCs for 24 h. As we can see in Figure 9A,B, all three green nanostructures showed good cytocompatibility to both IMR90 and MCF10A cells. Moreover, ZnO/ZrO_2_/rGO NCs displayed better cytocompatibility than pristine ZnO NPs. These parameters suggest that ZnO/ZrO_2_/rGO NCs demonstrated augmented anticancer activity and improved cytocompatibility compared to the pristine form of ZnO NPs. Bioactive phytochemicals present in ginger extract that mediated the green synthesis of ZnO NPs, ZnO/ZrO_2_ NCs, and ZnO/ZrO_2_/rGO NCs could prevent their side effects on normal cell lines.

### 3.8. Mechanism of Anticancer Activity

**Figure 10:**
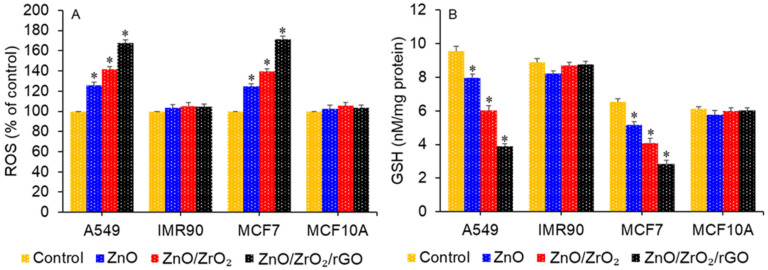
Effect of green synthesized nanostructures on oxidative stress parameters in cancer (A549 and MCF7) and normal (IMR90 and MCF10A) cells. (**A**) ROS level. (**B**) GSH level. * *p* < 0.05 vs. control.

Redox imbalance has been proposed as a possible pathway of the anticancer response of the nanostructured form of ZnO [4,36]. Intracellular ROS generation leads to depletion of the antioxidants (e.g., GSH) following exposure to appropriate concentrations of ZnO NPs, which causes oxidative damage in cancer cells. Conversely, in normal cells, this amount of ZnO NPs is unable to generate ROS above the threshold. This could be one of the promising mechanisms for selectively destroying malignant cells while producing minimal toxicity to non-cancerous normal cells [46,47]. Therefore, in this work, we further explored the possible pathways of anticancer activity of ZnO/ZrO_2_/rGO NCs through the examination of intracellular ROS and GSH levels in human cancer (A549 and MCF) and normal (IMR90 and MCF10A) cells. All the cells (cancerous and non-cancerous) were treated for 24 h with a moderate concentration (25 µg/mL) of green prepared nanostructures (ZnO NPs, ZnO/ZrO_2_ NCs, and ZnO/ZrO_2_/rGO NCs). Figure 10A,B showed elevated levels of ROS (pro-oxidant), whereas there were depleted levels of GSH in cancer cells following the treatment of ZnO NPs, ZnO/ZrO_2_ NCs, and ZnO/ZrO_2_/rGO NCs. Moreover, the effect of ZnO/ZrO_2_/rGO NCs on ROS generation and GSH depletion was higher than pristine form ZnO NPs.

**Figure 11:**
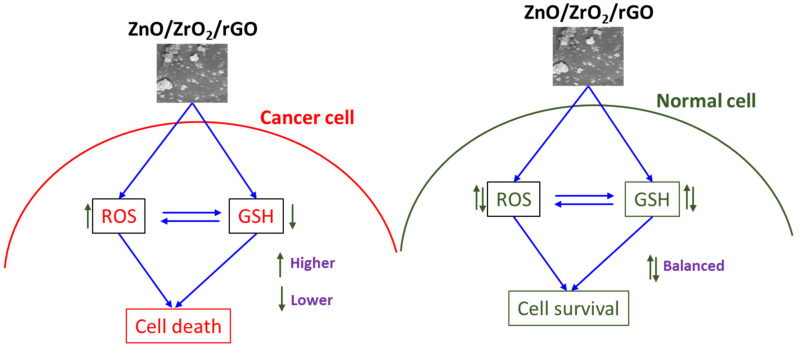
Role of oxidative stress in anticancer potential of green prepared ZnO/ZrO_2_/rGO NCs.

Oxidative stress-mediated possible mechanisms of anticancer activity of ZnO/ZrO_2_/rGO NCs are represented in Figure 11. The ZrO_2_ doping and rGO integration create two conclusive changes in the characteristics of ZnO NPs That plays an essential role in the augmented anticancer activity of ZnO/ZrO_2_/rGO NCs. First, ZrO_2_ and rGO addition decreases the particle size of ZnO. It is already reported that oxidative stress response nanostructures increase with the decreasing the size of the particles [48]. Second, PL results showed that ZrO_2_ and rGO reduce the PL spectral intensity of ZnO. Low spectral intensity of ZnO/ZrO_2_/rGO NCs suggests the recombination rate between electrons/holes pairs (e^−^/h^+^) was decreased due to the successful splitting of charges. This is an excellent phenomenon for surface redox reactions that can induce ROS generation and oxidative stress in biological systems. Conversely, green prepared ZnO NPs, ZnO/ZrO_2_ NCs, and ZnO/ZrO_2_/rGO NCs did not induce oxidative stress in non-cancerous normal (IMR90 and MCF10A) cells (Figure 11). This could be one of the possible mechanisms of the cytocompatibility of NPs/NCs prepared in this study.

## 4. Conclusions

Ginger (*Zingiber officinale*) rhizome extract was used for the facile, cost-effective, and environmentally friendly production of ZnO NPs, ZnO/ZrO_2_ NCs, and ZnO/ZrO_2_/rGO NCs. FETEM, FESEM, EDS, XRD, and PL investigations confirmed the preparation of ZnO NPs, ZnO/ZrO_2_ NCs, and ZnO/ZrO_2_/rGO NCs. Green prepared samples displayed excellent colloidal stability in aqueous suspension, as examined by DLS. Biological studies showed that ZnO/ZrO_2_/rGO NCs demonstrate 3–4 times higher anticancer efficacy in human cancer cells (A549 and MCF7) than the pristine form of ZnO NPs. In addition, ZnO/ZrO_2_/rGO NCs exhibit greater cytocompatibility in human normal cells (IMR90 and MCF10A) than those of pristine ZnO NPs. Higher anticancer efficacy and improved biocompatibility of ZnO/ZrO_2_/rGO NCs might be because the ginger extract mediated good synergism between ZnO, ZrO2 and rGO. The anticancer efficacy of ZnO/ZrO_2_/rGO NCs was also observed to be facilitated via oxidative stress, as evidenced by intracellular ROS generation and GSH depletion. Present results indicated that ginger extract mediated ZnO/ZrO_2_/rGO NCs could be a promising therapeutic drug in the treatment of cancer. This unique work warrants future research on the anticancer potential of ZnO/ZrO_2_/rGO NCs in different in vitro and in vivo models.

## Figures and Tables

**Table 1:** Physicochemical characterization of green prepared samples.

Parameters	ZnO NPs	ZnO/ZrO_2_ NCs	ZnO/ZrO_2_/rGO NCs
XRD size (nm)	25	20	13
TEM size (nm)	23	18	12
Hydrodynamic size (nm)	149	81	49
Zeta potential (mV)	−17	−21	−29

**Table 2:** IC_50_ values of green synthesized nanostructures.

Green Samples	A549 Cells	MCF7
ZnO NPs	40 µg/mL	47 µg/mL
ZnO/ZrO_2_ NCs	32 µg/mL	30 µg/mL
ZnO/ZrO_2_/rGO NCs	12 µg/mL	10 µg/mL

## Data Availability

The raw data will be available from the corresponding author upon reasonable request.

## Supplementary Materials

The following supporting information can be downloaded at: https://www.mdpi.com/article/10.3390/jfb14010038/s1. Figure S1. Endpoint chromogenic limulus amebocyte lysate (LAL) assay for endotoxin detection in prepared ZnO NPs, ZnO/ZrO2 NCs, and ZnO/ZrO2/rGO NCs. A concentration of 50 μg/ml of ZnO NPs, ZnO/ZrO2 NCs, and ZnO/ZrO2/rGO NCs were incubated with LAL (containing enzyme), or substrate, or both (LAL + substrate). After the completion of incubation time, absorbance of the substrate was measured at 405 nm. If endotoxin is present in the sample, a yellow color should develop only in the complete reaction mixture (NPs/NCs + LAL + substrate), not in other two mix-tures. The absorbance of the enzymatically cleaved p-nitroaniline part of the substrate peptide was measured at 405 nm by a microplate reader (Synergy-HT). Figure S2. DLS characterization of green prepared ZnO NPs, ZnO/ZrO2 NCs, and ZnO/ZrO2/rGO NCs. (A-C) Hydrodynamic size of ZnO NPs, ZnO/ZrO2 NCs, and ZnO/ZrO2/rGO NCs. (D-F) Zeta potential ZnO NPs, ZnO/ZrO2 NCs, and ZnO/ZrO2/rGO NCs.
