# Supplementary material for: Biosynthesis, Characterization, and Augmented Anticancer Activity of ZrO2 Doped ZnO/rGO Nanocomposite
Source: J Funct Biomater. 2023 Jan 9;14(1):38. doi: 10.3390/jfb14010038 (PMC9861721; doi:10.3390/jfb14010038)
Supplement: Supplementary file 1 [file jfb-14-00038-s001.zip › jfb-2046589-supplementary.pdf]

Supplementary Information

Biosynthesis, characterization, and augmented anticancer activity of ZrO_2 doped ZnO/rGO nanocomposite.

Maqusood Ahamed[1] *, Rashid Lateef[2], M.A. Majeed Khan[1], Pavan Rajanahalli[3], Mohd Javed Akhtar[1]

[1]King Abdullah Institute for Nanotechnology, King Saud University, Riyadh 11451, Saudi Arabia

[2]Department of Biochemistry, Faculty of Science, Veer Bahadur Singh Purvanchal University, Jaunpur 222003, Uttar Pradesh, India

[3]Department of Biology, University of Tampa, Tampa, FL 33569, USA

*Correspondence: mahamed@ksu.edu.sa (Maqusood Ahamed)

…………………………………………………………………………………………

Endpoint chromogenic limulus amebocyte lysate (LAL) assay

The endpoint chromogenic limulus amebocyte lysate (LAL) assay kit (Lonza, Basel, Switzerland) was used to examine endotoxin contamination in ZnO NPs, ZnO/ZrO_2 NCs, and ZnO/ZrO_2/rGO NCs. This assay has a sensitivity range of 0.1 EU/ml - 1.0 EU/ml. Briefly, ZnO NPs, ZnO/ZrO_2 NCs, and ZnO/ZrO_2/rGO NCs at a concentration of 50 μg/ml was were mixed with the LAL supplied in the test kit and incubated at 37 °C for 10 min. A peptide substrate solution was then mixed with the LAL-sample mixture and incubated at 37 °C for next 6 min. The reaction was then stopped by addition of stop reagent supplied with the kit. In addition to the complete reaction mixture (i.e. NPs/NCs+LAL+substrate), two additional mixtures were prepared to check the possible interference of NPs/NCs in the assay, namely NPs/NCs+LAL and BT NPs+substrate. If endotoxin is present in the sample, a yellow color should develop only in the complete reaction mixture, not in other two mixtures. The absorbance of the enzymatically cleaved p-nitroaniline part of the substrate

peptide was measured at 405 nm in a microplate reader (Synergy-HT, BioTek, Vinnoski, VT, USA). Since this absorbance is in direct proportion to the amount of endotoxin present, the concentration of endotoxin can be calculated from a standard curve using LPS.

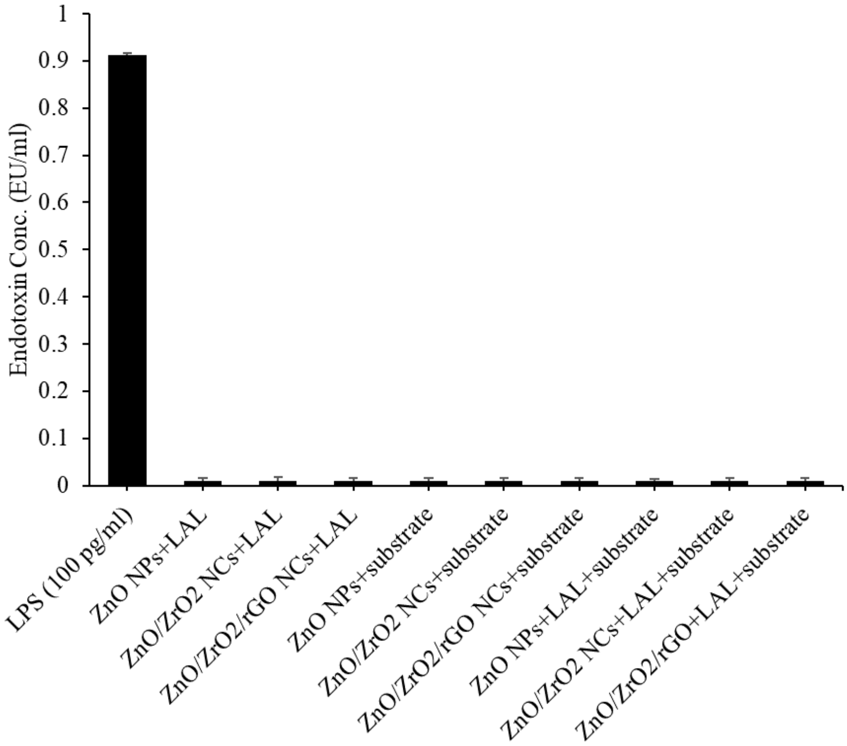

Figure S1. Endpoint chromogenic limulus amebocyte lysate (LAL) assay for endotoxin detection in prepared ZnO NPs, ZnO/ZrO_2 NCs, and ZnO/ZrO_2/rGO NCs. A concentration of 50 μg/ml of ZnO NPs, ZnO/ZrO_2 NCs, and ZnO/ZrO_2/rGO NCs were incubated with LAL (containing enzyme), or substrate, or both (LAL+substrate). After the completion of incubation time, absorbance of the substrate was measured at 405 nm. If endotoxin is present in the sample, a yellow color should develop only in the complete reaction mixture (NPs/NCs+LAL+substrate), not in other two mixtures. The absorbance of the enzymatically cleaved p-nitroaniline part of the substrate peptide was measured at 405 nm by a microplate reader (Synergy-HT).

Results showed that there is no endotoxin contamination in synthesized ZnO NPs, ZnO/ZrO_2 NCs, and ZnO/ZrO_2/rGO NCs.

Hydrodynamic size and zeta potential examination

Hydrodynamic size and zeta potential of green prepared ZnO NPs, ZnO/ZrO_2 NCs, and $ZnO/ZrO_2/rGO$ NCs was examined in culture media (DMEM) (Figure S2).

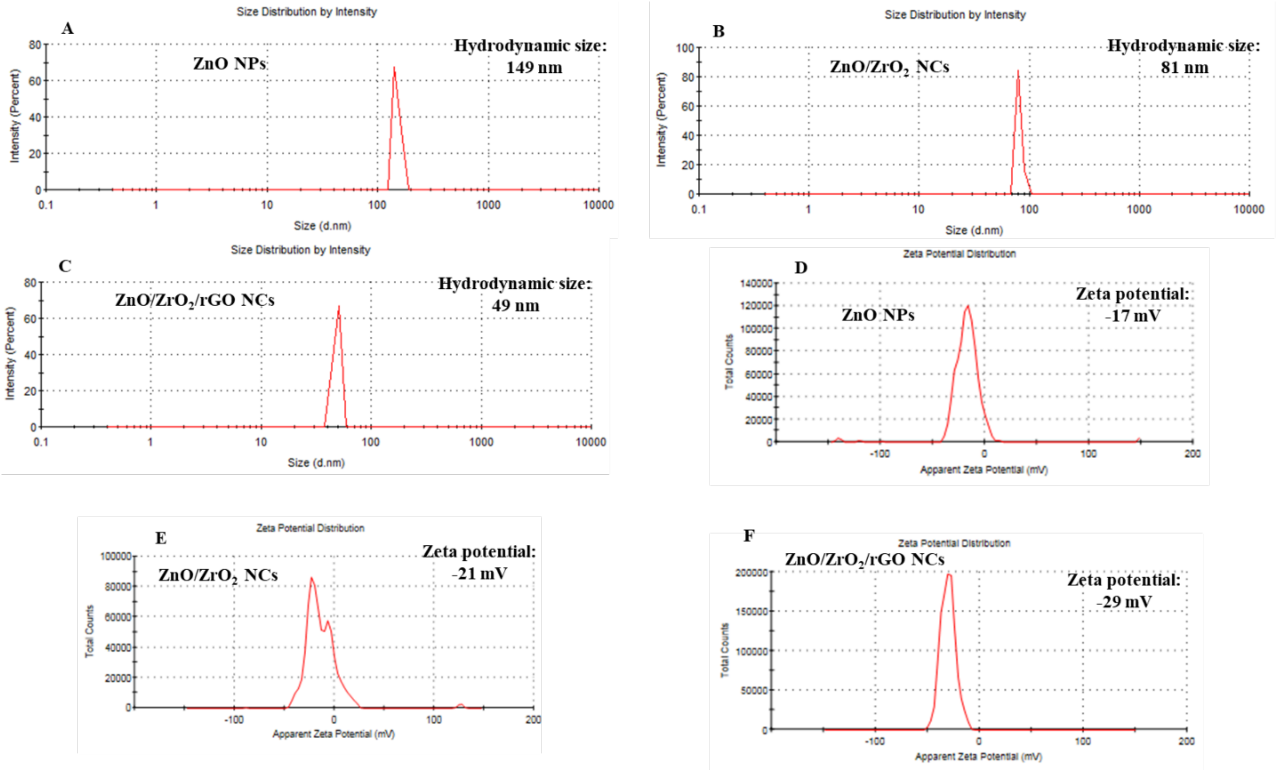

Figure S2. DLS characterization of green prepared ZnO NPs, ZnO/ZrO_2 NCs, and $ZnO/ZrO_2/rGO$ NCs. (A-C) Hydrodynamic size of ZnO NPs, ZnO/ZrO_2 NCs, and $ZnO/ZrO_2/rGO$ NCs. (D-F) Zeta potential ZnO NPs, ZnO/ZrO_2 NCs, and $ZnO/ZrO_2/rGO$ NCs.
